# A new ultrasound‐guided surgical technique to fix acute tibial posterior cruciate ligament avulsion fracture

**DOI:** 10.1002/jeo2.70191

**Published:** 2025-02-28

**Authors:** Hao Luo, Lin‐Feng Li, Song Han, Yu Pan, Fei‐Ju Xu, Tao Liu

**Affiliations:** ^1^ Department of Orthopaedic Surgery The Affiliated People's Hospital of Jiangsu University Zhenjiang Jiangsu China; ^2^ Department of Orthopaedic Southwest Hospital Jiangbei Area (The 958th Hospital of Chinese People's Liberation Army) Chongqing Sichuan China; ^3^ Department of Ultrasound Medicine The Affiliated People's Hospital of Jiangsu University Zhenjiang Jiangsu China

**Keywords:** Adjustable‐loop cortical button, Posterior cruciate ligament, Suspensory fixation, Tibial avulsion fracture, Ultrasound

## Abstract

**Purpose:**

This study aims to describe a novel minimally invasive technique for the treatment of acute tibial posterior cruciate ligament (PCL) avulsion fracture.

**Methods:**

This retrospective study included seven patients who underwent ultrasound‐guided fixation for acute PCL tibial avulsion fractures by using an adjustable‐loop device between January 2021 and January 2023. Before the surgery, the maximum diameter, area and displacement distance of the fragments were measured using computed tomography examination. All patients were followed up for at least 12 months, and clinical outcomes were assessed on the basis of range of motion, the International Knee Documentation Committee Score and the Lysholm score.

**Results:**

For the seven patients, the mean maximum diameter, area and displacement distance of preoperative avulsion fragments were 12.7 mm (range, 9.0–48.3), 128 mm^2^ (range, 63–256.2) and 5.9 mm (range, 3.8–7.2), respectively. These fractures were fixed using an adjustable‐loop suspensory device under ultrasound guidance. Based on x‐ray examination during the post‐operative follow‐up period, all patients had no fracture displacement and fracture unions were confirmed, with a mean union time of 10.28 ± 2.13 weeks (range, 8–14). Based on the knee function assessment at 12‐month post‐operative follow‐up visit, all patients demonstrated excellent clinical outcomes.

**Conclusions:**

Ultrasound‐assisted internal fixation using an adjustable‐loop device demonstrated satisfactory clinical and radiographic results. This technique has the advantages of being minimally invasive, safe, stable, convenient to operate and thus could be considered as a feasible alternative for the treatment of acute tibial PCL avulsion fractures.

**Level of Evidence:**

Level III.

## Graphical abstract

Acute tibial posterior cruciate ligament avulsion fracture can be fixed by the ultrasound‐guided surgical technique using an adjustable‐loop device, with the advantages of being minimally invasive, safe and stable.

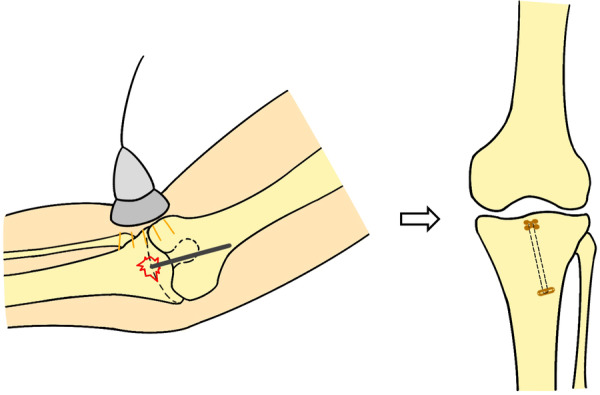

**Abbreviations**
3D: three‐dimensional
ARIF: arthroscopic reduction and internal fixation
CT: computed tomography
IKDC: International Knee Documentation Committee
K‐wire: Kirschner wire
MRI: magnetic resonance imaging
ORIF: open reduction and internal fixation
PCL: posterior cruciate ligament
PDS: polydioxanone
ROM: range of motion

## INTRODUCTION

Posterior cruciate ligament (PCL) is the primary restraint mechanism to prevent backward displacement of tibia, and it works alongside the anterior cruciate ligament to maintain the stability of the knee joint [9]. When the knee is flexed or hyperextended and subjected to high‐energy impact from the front to back, the tension on PCL can exceed its tolerance limit, resulting in injuries such as PCL tibial avulsion fractures [17]. These fractures usually require surgical intervention to achieve anatomical reduction and restore the appropriate tension of the ligament [7, 13, 19]. Improper treatment can lead to complications such as fracture non‐union and knee joint instability [8, 14, 25]. However, orthopaedic surgeons face considerable challenges when fixing PCL tibial avulsion fractures, due to the deep location of PCL and the complex anatomy of the surrounding structures.

Currently, no universally accepted optimal surgical method is available for treating tibial avulsion fractures of PCL. The traditional open surgery requires a large incision, which provides excellent exposure to the fracture, thus facilitating the operation and reduction process [10]. However, it is invasive and often causes increased post‐operative pain and noticeable scarring. Arthroscopic surgery has gained popularity owing to its potential for minimal invasion and faster recovery, and it allows for the repair of fragments and other soft tissue injuries under direct vision, which can achieve favourable treated outcomes [4, 11]. However, arthroscopic suture fixation is intricate and necessitates extensive training and advanced surgical skills, thereby limiting its wide clinical application.

This study introduces a novel technique for treating PCL tibial avulsion fractures using an adjustable‐loop device under B‐ultrasound guidance to further minimise invasive procedures and reduce the risk of related complications.

## METHODS

**Figure 1:**
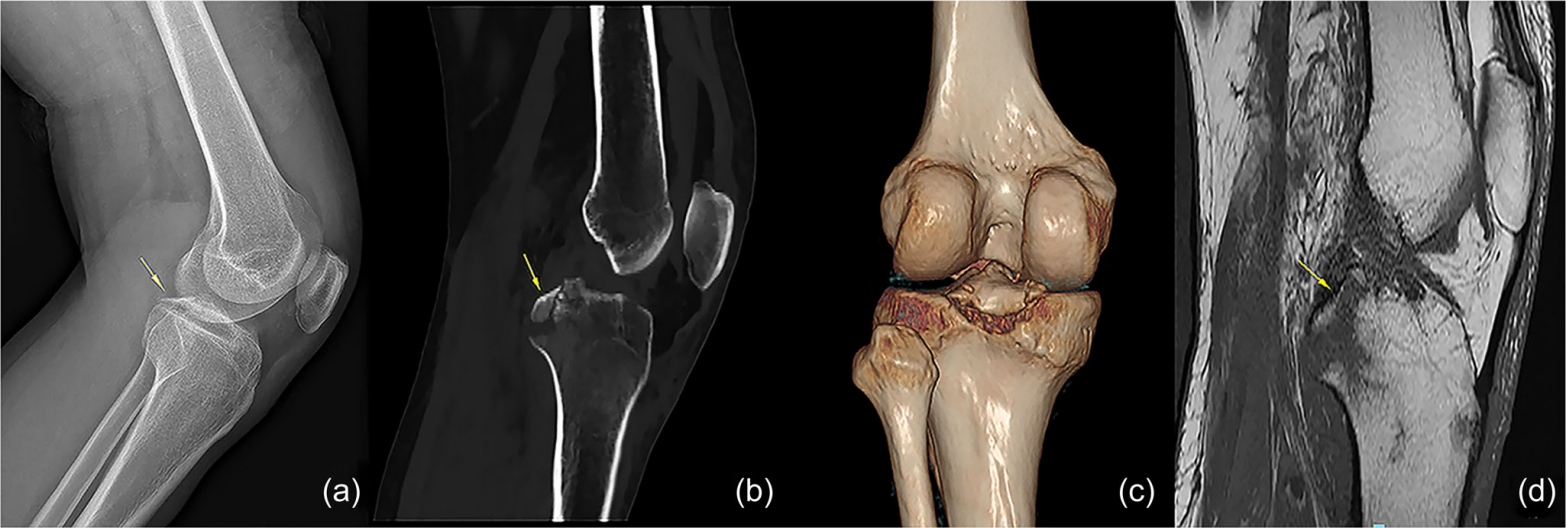
Preoperative imaging of PCL tibial avulsion fractures. (a) Knee x‐ray, (b) sagittal section scan and (c) 3D CT scan reconstruction showed slightly proximally displaced avulsion fracture of PCL. (d) MRI demonstrated loose PCL with tibial attachment slightly displaced proximally. The yellow arrows show the avulsion fragment. 3D, three‐dimensional; CT, computed tomography; MRI, magnetic resonance imaging; PCL, posterior cruciate ligament.

This study received approval from the Ethics Committee. The procedures used in this study follow the principles of the Declaration of Helsinki. The new surgical approach was implemented to treat acute PCL tibial avulsion fractures in seven patients between January 2021 and January 2023. The inclusion criteria for this study were as follows: (1) intact knee function prior to injury, (2) acute or subacute trauma to the knee (time of injury less than 3 weeks), (3) positive posterior drawer test and (4) radiographical image indicating isolated PCL avulsion fracture. The exclusion criteria included the following: (1) obesity (body mass index >30), (2) old fractures, (3) comminuted fractures or small bone fragments (minimum transverse diameter <8 mm), (4) excessive displacement (>20 mm) and (5) combined with ligament rupture. X‐rays and three‐dimensional (3D) computed tomography (CT) scan reconstruction were applied preoperatively to assess the size and displacement of the fragment (Figure 1a–c). The tension and integrity of PCL were examined using magnetic resonance imaging (MRI) (Figure 1d). All patients provided informed consent to accept this surgical technique. All the operations were performed by a senior orthopaedic surgeon (TL), assisted by a sonographer (FJX).

### Surgical technique

**Figure 2:**
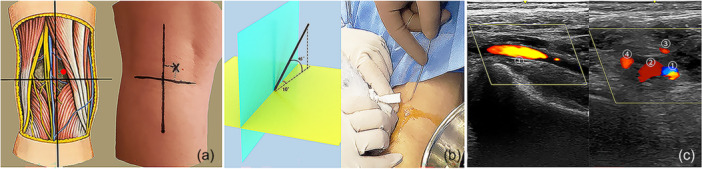
Ultrasound‐assisted positioning for insertion of K‐wire. (a) Entry point of wire was 1 cm each away from vertical and horizontal axes in the medial‐upper quadrants. (b) K‐wire was placed at angles of 10° in reference to the sagittal plane (blue) and 45° in reference to the coronal plane (yellow). (c) Neurovascular structures were identified in the sagittal and transverse plane under B‐ultrasound monitoring: (1) popliteal artery, (2) popliteal vein, (3) medial inferior genicular vein and (4) lateral inferior genicular vein. K‐wire, Kirschner wire.

**Figure 3:**
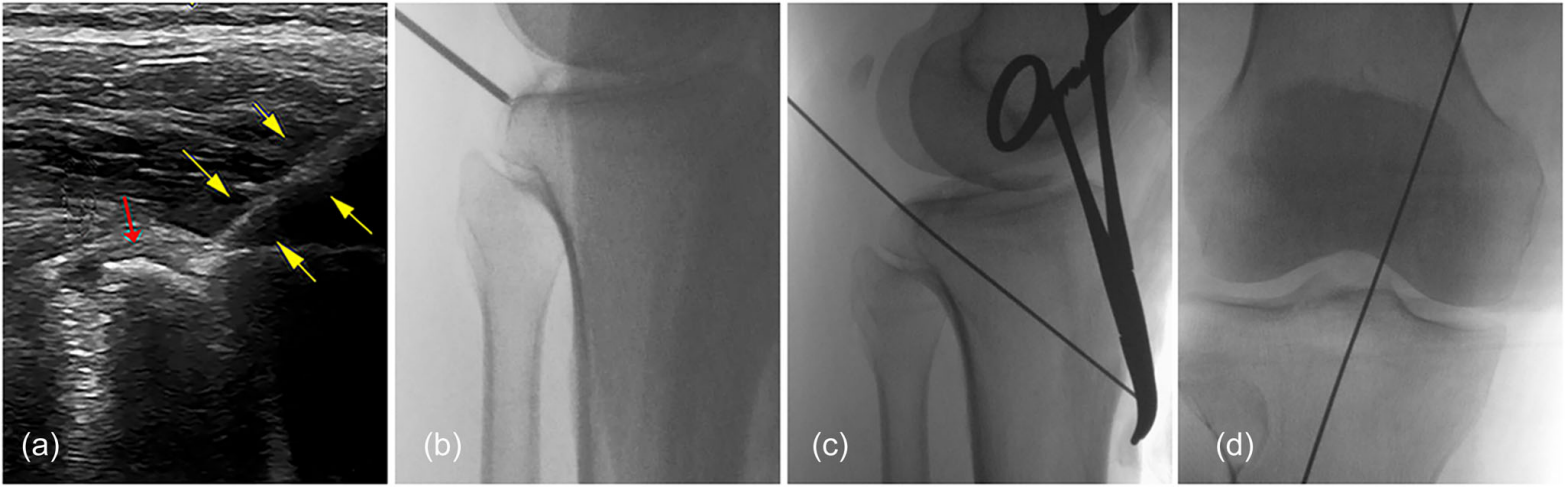
Intraoperative reduction in the fracture and insertion of K‐wire. (a) Avulsion fragment (as illustrated with red arrowhead) was reduced with K‐wire (as illustrated with yellow arrowheads) under B‐ultrasound guidance. (b) Image intensifier showed the fragment and the K‐wire were both in accepted position. (c, d) Lateral and anteroposterior views showed that the wire was drilled through the contralateral tibial cortex. K‐wire, Kirschner wire.

**Figure 4:**
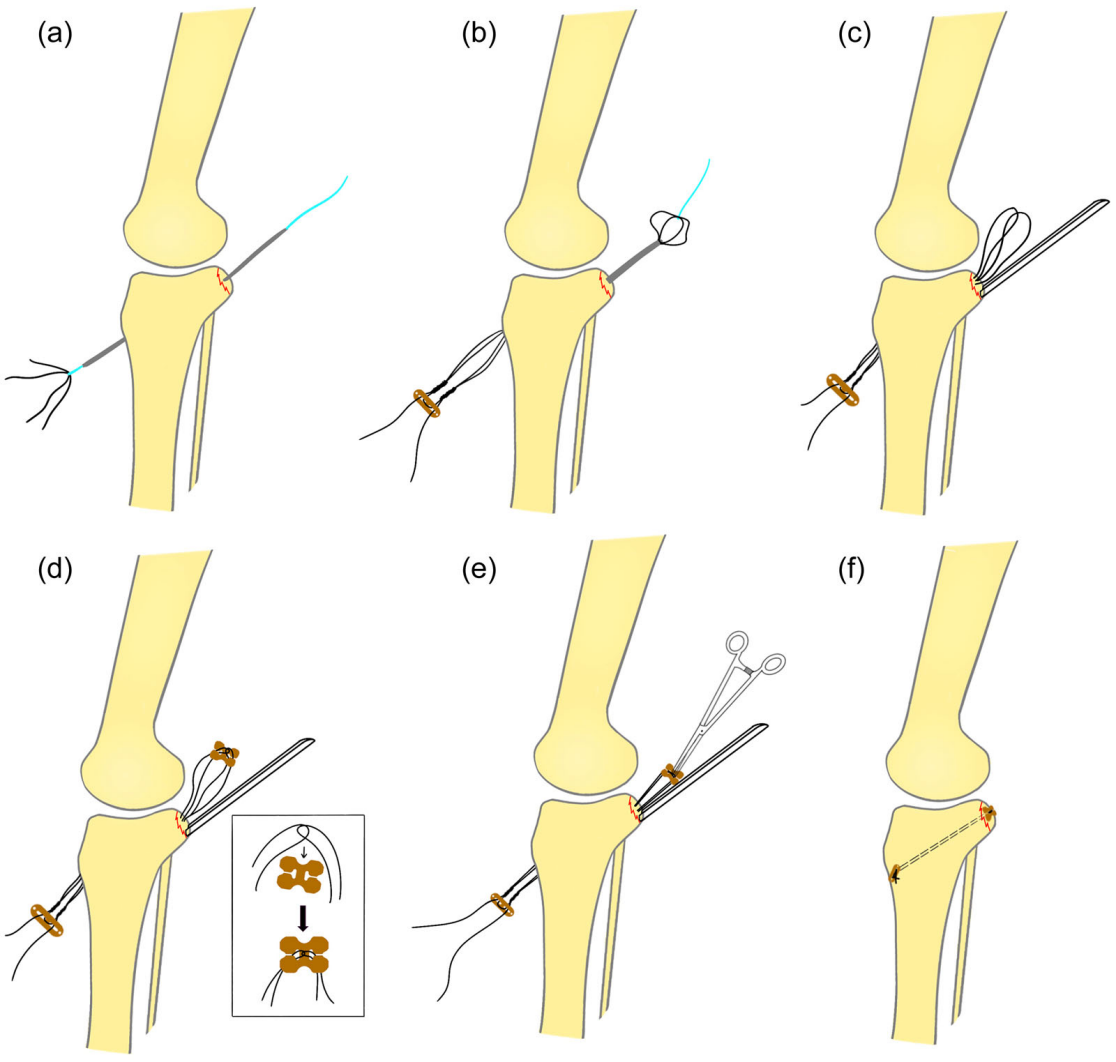
Fixation of the PCL avulsion fragment with suspensory devices. (a) PDS suture was introduced through the cannulated drill and its end is tied with adjustable‐loop. (b) The adjustable‐loop was passed through the tibial tunnel by pulling PDS suture rearward. (c) Semi‐tubular chute was inserted along the medial side of the sleeve and replaced for subsequent procedures. (d) AC clover‐shaped titanium plate was mounted on the adjustable‐loop at the posterior. (e) AC clover‐shaped titanium plate was inserted along the semi‐tubular chute, with the traction of the adjustable‐loop at the anterior. (f) Adjustable‐loop cortical button was tightened and knotted at the anterior cortex of the tibia. PCL, posterior cruciate ligament; PDS, polydioxanone.

The surgical procedure was conducted under general anaesthesia, with the patient positioned prone. The affected knee was maintained at 20° of flexion, and a thigh tourniquet was applied. After sterilisation was conducted and the operating sheet was laid out, the ultrasound (LOGIQ‐E9) probe was wrapped with aseptic plastic film for later intraoperative use. For precise positioning, the popliteal fossa was divided into four quadrants by setting the popliteal crease as the horizontal axis and the midline of popliteal fossa as the vertical axis. The entry point was determined to be 1 cm away from the vertical and horizontal axes in the medial‐upper quadrant (Figure 2a). A 2.5 mm Kirschner wire (K‐wire) was percutaneously inserted at the centre of the fragment at the angles of 10° relative to the sagittal plane and 45° relative to the coronal plane of tibia (Figure 2b). Ultrasound was used to monitor neurovascular structures, ensuring that they were at a safe distance from the K‐wire during the insertion process (Figure 2c). After the fragment was touched with the K‐wire under B‐ultrasound guidance, it was reduced and temporarily fixed by tapping the K‐wire gently (Figure 3a). An image intensifier was used to confirm that the fragment and the K‐wire were in the accepted position (Figure 3b). Then, the K‐wire was drilled forward through the contralateral tibial cortex and skin (Figure 3c,d). Two 10–12 mm width skin incisions were made at the posterior insertion and anterior exit points of the K‐wire. Sleeves with gradually increasing calibres were sequentially inserted along the K‐wire to the surface of the fragment for blunt dissection, until the sleeve with a calibre of 10 mm in width was applied, facilitating the subsequent insertion of fixative devices and avoiding the possibility of neurovascular damage. A 3.5 mm cannulated drill was used to build a tibial tunnel along the wire through the anterior cortex of the tibia and skin under sleeve protection. After the K‐wire was removed, a No. 1 polydioxanone (PDS) suture was threaded from the anterior to the posterior through the cannulated drill, and its end was tied with the loop of an adjustable‐loop cortical button (ETButton REJOIN, size of 12 mm × 4 mm × 1.2 mm) at the anterior to the tibia (Figure 4a). The cannulated drill was pulled out using a plier, and the loop was passed through the tibial tunnel by pulling the suture rearward, with the cortical button left at the anterior to the tibia. During this process, the sleeve was left unmoved to prevent fragment displacement (Figure 4b). Before the sleeve was removed, a semi‐tubular chute was inserted along the medial side of the sleeve, to insert a titanium plate later and avoid embedding of the neurovascular structures and other soft tissues (Figure 4c). Then, an AC clover‐shaped titanium plate (ETButton REJOIN, size of 10 mm × 8 mm × 1.3 mm) was mounted at the end of the adjustable‐loop in the rear (Figure 4d). Subsequently, the AC clover‐shaped plate was inserted using straight clamp along the chute, with the traction of the adjustable‐loop at anterior to the tibia, until it touched the fragment (Figure 4e). The adjustable‐loop cortical button was tightened to ensure that the fragment was compressed, and the loop was knotted at the anterior cortex of the tibia (Figure 4f). During the whole process of inserting the clover‐shaped plate, ultrasound was used to confirm that neurovascular structures or other soft tissue were not embedded by the clover‐shaped plate. The image intensifier was used to confirm that the position of the clover‐shaped titanium plates and fragments was satisfactory, and the range of motion (ROM) of the knee joint was checked as normal. Finally, the thread was cut, and the incisions were sutured.

### Post‐operative treatment and rehabilitation

After the surgery, the knee was dressed with sterile cotton bandages and the affected limb was immobilised in an extension position with a brace. Post‐operative functional exercises and physical therapy were directed by a rehabilitation physician. Quadriceps isometric exercises can be started under brace fixation on the first day post‐operatively. Passive knee flexion and extension was performed 3 days after the surgery. The patients were allowed to support part of the weight with crutches 3 weeks post‐operatively. The brace can be immobilised, and active flexion and extension can be performed at 4 weeks post‐operatively. Full weight‐bearing walking was started 8 weeks post‐operatively, and preoperative situation can be recovered 12 weeks after surgery.

### Post‐operative follow‐up

Post‐operative follow‐ups were conducted once a week until the sutures were taken out. Then, the patients were followed up at post‐operative 4 weeks, 6 weeks, 2 months and 3 months and every month thereafter until 12 months. They were followed up at least once a quarter from 1 year post‐operatively. During the follow‐up visits, x‐ray examination was performed regularly every month to observe whether the avulsion fracture achieved radiological union. The ROM of the knee was measured using a traditional goniometer to reflect the movability of the knee. The posterior drawer test was used to evaluate knee laxity after intraoperative fixation and at each follow‐up visit after 6 weeks post‐operatively. It was identified as positive if the tibia of the affected limb can be pushed backwards by more than 5 mm or if it moves significantly compared with the contralateral normal knee. The function of the affected limb was evaluated using the International Knee Documentation Committee Score (IKDC)‐Subjective Knee Form scale and the Lysholm scale. All follow‐up and clinical evaluations are performed by a senior orthopaedic surgeon (TL). The ROM of the knee joint, the IKDC score, and the Lysholm score at 12 months after surgery were analysed to evaluate the recovery of the affected knee.

### Statistical analysis

SPSS (version 27.0) was used to analyse data, and *t* test was used to compare the pre‐ and post‐operative knee ROMs, Lysholm scores and IKDC scores. All measurement data were expressed as the mean ± standard deviation. *p* < 0.05 indicated statistical significance between the two groups, and it was used to evaluate the curative effect of the new technology.

## RESULTS

This study included seven patients in total, including five males and two females, with an average age of 40 years (range, 26–51 years). Amongst them, five patients sustained injuries from traffic accidents, one from a sport‐related accident, and one from a fall. Based on the measurements of 3D CT reconstruction, the average maximum diameter of the avulsion fragment was 12.7 ± 2.94 mm (range, 9.0–48.3), the average area was 128 ± 65.23 mm^2^ (range, 63–256.2) and the average displacement distance was 5.9 ± 1.21 mm (range, 3.8–7.2 mm). Detailed patient characteristics and measurements of the PCL avulsion fragments are available in Tables S1 and S2. All the operations were successfully completed, and no complications were encountered, including neurovascular injury and infection. All patients had a negative result on the posterior drawer test with a hard endpoint after intraoperative fixation.

**Figure 5:**
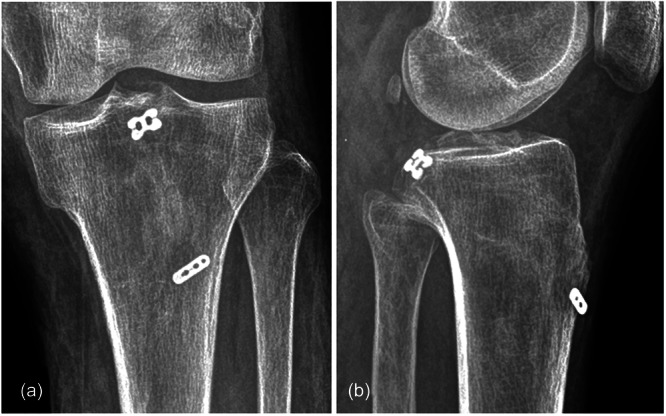
Post‐operative plain radiographs showing fracture union with adjustable‐loop suspensory device. (a) Anteroposterior and (b) lateral views of the knee are shown.

The follow‐up duration was 12–30 months, with an average of 17.1 months. Radiographic examination confirmed satisfactory reduction and fracture union in all patients (Figure 5), with an average union time of 10.28 ± 2.14 weeks (range, 8–14 weeks). The posterior drawer tests of all patients were negative at each follow‐up visit after 6 weeks post‐operatively. All the follow‐up patients were satisfied with the recovered flexion and extension function in the affected knee at 3 months post‐operatively. The mean knee ROM increased from 31.43 ± 19.52° (range, 10–70°) in preoperative to 125.71 ± 9.32° (range, 110–135°) in post‐operative 12 months (*p* < 0.05). The Lysholm scores of the affected knee were 30.71 ± 11.43 points (range, 18–47) in preoperative and 94.86 ± 2.34 points (range, 92–98) in post‐operative 12 months (*p *< 0.05). The IKDC scores were 32.86 ± 8.09 (range, 20–45) and 76.71 ± 6.07 (range, 69–86) points on preoperative and post‐operative 12 months, respectively (*p* < 0.05), as presented in Table 1. Detailed data of preoperative and 12 months post‐operative knee function assessment are available in Table S3.

**Table 1:** Comparison of preoperative and 12 months post‐operative knee function assessment.

Variables	Preoperative	12 months post‐operative	*p*
ROM (deg)	31.43 ± 19.52	125.71 ± 9.32	0.001*
Lysholm score (points)	30.71 ± 11.43	94.86 ± 2.34	0.001*
IKDC score (points)	32.86 ± 8.09	76.71 ± 6.07	0.001*

*Note*: Continuous variables are presented as means ± standard deviations.

Abbreviations: deg, degree; IKDC, International Knee Documentation Committee; ROM, range of motion.

* Indicates statistical significance (*p* < 0.05).

## DISCUSSION

In this study, a novel ultrasound‐guided surgical technique for the fixation of acute PCL avulsion fracture by using an adjustable‐loop device is described. The technique combines the advantages of minimal invasiveness, safety, convenience and stable fixation. It showed satisfactory clinical and radiographic outcomes during the follow‐up.

Previous studies showed that PCL tibial avulsion fractures can be treated by open reduction and internal fixation (ORIF) and arthroscopic reduction and internal fixation (ARIF). Conventional ORIF surgical incisions are made between the inner and outer head of the gastrocnemius muscle, and hysteresis screws are used to fix the fragment, achieving satisfactory reduction results [16]. However, significant trauma and scarring have prompted surgeons to explore minimally invasive alternatives [21]. Gavaskar et al. described a short transverse incision of 3 or 4 cm in the popliteal fossa to further reduce invasiveness [5]. This incision utilises the plane between the two heads of the gastrocnemius muscle and exposes the fracture area by blunt dissection. Despite precise preoperative planning and localisation, small incisions provide limited visualisation, which may heighten the risk of damage to neurovascular structures. Abdallah and Arafa reported an L‐shaped posteromedial incision that protects vascular nerve structures and exposes the joint capsule by bluntly separating the medial cephalic of the gastrocnemius and semimembranosus muscles and stretching the gastrocnemius muscle outward [1]. This incision enables direct visualisation of the fragment without exposing nearby blood vessels and nerves. However, this method complicates the visualisation of the lateral base of the avulsed tibial fragment and makes it challenging to secure internal fixation perpendicular to the fracture plane, which ultimately affects the stability of internal fixation [3]. Compared with ORIF, the ultrasound‐guided surgical technique in the present study minimises invasiveness, thus significantly reducing soft tissue destruction and scarring. This technique involves an adjustable‐loop only through the tibial tunnel, which greatly reduces damage to the tibia.

In recent years, various arthroscopic fixation techniques, including the combination of canulated screws, sutures, adjustable length rings, intimal buttons and bioresorbable anchors, have been widely reported [2, 6, 15, 18, 22, 24]. Arthroscopic techniques are less invasive than open‐reduction surgeries and allow for reducing fractures under direct visualisation, which has a more desirable reduction effect when treating avulsion fractures of small fragments. However, manipulation of the posterior mediastinum may jeopardise posterior neurovascular structures, and passing cross‐sutures through the PCL bundle may injure or sever ligament [6, 23]. When establishing a tibial tunnel, thorough debridement of the tibial bed is necessary until the posterior distal edge of the bone bed is clearly visible, which may affect the blood supply to the fragments. Arthroscopic techniques are relatively complex and challenging, requiring extensive training and advanced arthroscopic techniques, which may not be friendly to many orthopaedic surgeons [12]. The ultrasound‐guided technique demonstrated in the present study requires less operational expertise and specialised surgical equipment than ARIF, thereby decreasing the difficulty and duration of the operation. Ultrasound is easy to operate and can be performed without radiation exposure. It can be effectively used to identify vascular and nerve structures during minimally invasive surgery, thus significantly decreasing the risk of neurovascular damage. In addition, the avulsion fracture was fixed using an adjustable‐loop suspension with a clover‐shaped titanium plate that provides a larger contact surface with the fragment to decrease the risk of being cut or chipped, thereby enhancing the stability of the fixation.

This study demonstrates that ultrasound‐guided fixation using adjustable‐loop devices can be applied to effectively repair most acute PCL avulsion fractures occurring as large, isolated fragments, achieving satisfactory reduction and fracture healing. A previous study has shown that a button plate can be used for the fixation of comminuted PCL avulsion fractures [20]. A notable detail is that the clover‐shaped titanium plate has the potential to achieve effective fixation in the case of comminuted fractures due to its large area of contact with the fragment, which allows it to grasp and compress the entire PCL attachment. Future studies should focus on the efficacy of this new surgical technique in comminuted cases.

This study has some shortcomings. First, this study is a retrospective study with a small sample size, which may lead to selection and recall biases that may have caused deviations in the evaluation of the surgical effects. Second, this surgical technique requires the cooperation of one sonologist and one radiologist. Third, this technique is not indicated for patients with excessive displacement or combined ligament rupture.

## CONCLUSIONS

A novel surgical technique is demonstrated in this study for the effective fixation of PCL tibial avulsion fractures by using an adjustable‐loop device under ultrasound guidance, showing satisfactory clinical and radiographic results. The inserted point and orientation of the K‐wire in this method can be set to standardise and refine the surgical procedure. This technique has the advantages of being minimally invasive, safe and stable, and it can be performed in most trauma surgeries, thus deserving further promotion as a feasible alternative for the treatment of acute tibial PCL avulsion fractures.

## AUTHOR CONTRIBUTIONS

Hao Luo designed the study and performed writing—original draft and review. Lin‐Feng Li participated in original drafting and review. Song Han and Yu Pan performed data collection and analysis. Fei‐Ju Xu participated in the study design and performed intraoperative ultrasound manipulation. Tao Liu proposed the new technique, was the main surgeon, carried out the post‐operative visits and performed writing—review and editing. All authors read and approved the final manuscript.

## CONFLICT OF INTEREST STATEMENT

The authors declare no conflicts of interest.

## ETHICS STATEMENT

This study was approved by the Institutional Ethics Committee of The Affiliated People's Hospital of Jiangsu University (SQK‐20220014‐Y) and complied with the Declaration of Helsinki. Informed consent was obtained from all the participants.

## Supporting information

Supporting information.

## Data Availability

The data that support the findings of this study are available on reasonable request from the corresponding author.
