# Supplementary material for: A new ultrasound‐guided surgical technique to fix acute tibial posterior cruciate ligament avulsion fracture
Source: J Exp Orthop. 2025 Feb 28;12(1):e70191. doi: 10.1002/jeo2.70191 (PMC11869579; doi:10.1002/jeo2.70191)
Supplement: Supplementary file 1 — Supporting information. [file JEO2-12-e70191-s001.docx]

**Supporting Information**

**Supplementary Table S1.** Patients’ Characteristics

| No. | gender | age (years) | height (m) | weight (kg) | BMI | cause | Follow-up time (months) | Time of fracture union (weeks) |
| --- | --- | --- | --- | --- | --- | --- | --- | --- |
| 1 | male | 26 | 1.72 | 70 | 25.35 | traffic accident | 12 | 10 |
| 2 | male | 35 | 1.67 | 65 | 24.02 | traffic accident | 12 | 8 |
| 3 | male | 36 | 1.75 | 75 | 22.86 | athletics | 18 | 8 |
| 4 | male | 48 | 1.65 | 70 | 25.71 | traffic accident | 12 | 10 |
| 5 | male | 51 | 1.7 | 72 | 24.91 | fall | 24 | 12 |
| 6 | female | 39 | 1.6 | 55 | 21.48 | traffic accident | 30 | 14 |
| 7 | female | 45 | 1.58 | 50 | 24.03 | traffic accident | 12 | 10 |

Abbreviations: BMI, Body Mass Index.

**Supplementary Table S2.** Measurement Data of the PCL avulsion fragments

| No. | maximum diameter (mm) | area (mm^2^) | displacement (mm) |
| --- | --- | --- | --- |
| 1 | 9 | 63 | 5 |
| 2 | 13 | 117 | 6.4 |
| 3 | 11.5 | 86 | 3.8 |
| 4 | 18.3 | 256.2 | 5.5 |
| 5 | 10.8 | 94 | 7 |
| 6 | 12.6 | 113 | 6.4 |
| 7 | 14 | 168 | 7.2 |
| Mean | 12.74 | 128.17 | 5.9 |
| SD | 2.94 | 65.23 | 1.21 |

Abbreviations: PCL, posterior cruciate ligament; SD, standard deviation.

**Supplementary Table S3.** The Data of preoperative and 12 months postoperative knee function assessment

| No. | Preoperative ROM (deg) | 12-Month postoperative ROM (deg) | Preoperative Lysholm score | 12-Month postoperative Lysholm score | IKDC Score | 12-month IKDC Score |
| --- | --- | --- | --- | --- | --- | --- |
| 1 | 20 | 120 | 20 | 92 | 30 | 80 |
| 2 | 30 | 130 | 35 | 94 | 30 | 78 |
| 3 | 40 | 135 | 42 | 97 | 40 | 80 |
| 4 | 70 | 135 | 47 | 98 | 45 | 86 |
| 5 | 20 | 120 | 21 | 95 | 20 | 70 |
| 6 | 10 | 110 | 18 | 92 | 30 | 69 |
| 7 | 30 | 130 | 32 | 96 | 35 | 74 |
| Mean | 31.43 | 125.71 | 30.71 | 94.86 | 32.86 | 76.71 |
| SD | 19.52 | 9.32 | 11.43 | 2.34 | 8.09 | 6.07 |

Abbreviations: SD standard deviation, deg degree, ROM range of motion, IKDC International Knee Documentation Committee Score.
